# ﻿Two new species of the genus *Neoproutista* Yang & Wu, 1994 (Hemiptera, Fulgoroidea, Derbidae) from southern China, with notes on their host plants

**DOI:** 10.3897/zookeys.1244.152550

**Published:** 2025-07-08

**Authors:** Sha-Sha Lv, Yong-Jin Sui, Lin Yang, Yu-Bo Zhang, Nian Gong, Xiang-Sheng Chen

**Affiliations:** 1 Institute of Entomology, Guizhou University, Guiyang, Guizhou, 550025, China; 2 The Provincial Special Key Laboratory for Development and Utilization of Insect Resources of Guizhou, Guizhou University, Guiyang, Guizhou, 550025, China; 3 College Agriculture, Anshun University, Anshun, Guizhou, 561000, China; 4 Guizhou Provincial Engineering Research Center of Medical Resourceful Healthcare Products, Guiyang Healthcare Vocational University, Guiyang, Guizhou, 550081, China

**Keywords:** checklist, derbids, distribution, Fulgoromorpha, identification key, Oriental region, planthoppers, taxonomy

## Abstract

Two new species of the genus *Neoproutista* Yang & Wu, 1994 (Hemiptera: Fulgoroidea: Derbidae) are described from southern China: *N.lobata* Lv, Sui & Chen, **sp. nov.** from Guizhou Province, and *N.quinaria* Lv, Sui & Chen, **sp. nov.** from Fujian Province. Detailed morphological descriptions, illustrations, and diagnostic comparisons are provided for both new species, with a particular emphasis on the male genital structures that distinguish them from congeners. A revised identification key to all known species of *Neoproutista* is also presented. A possible host plant is reported for the first time. The discovery of these taxa expands the known diversity and distribution of the genus in the Oriental region, underscoring the richness of derbid planthoppers in subtropical China.

## ﻿Introduction

The derbid genus *Neoproutista* was established by Yang and Wu (1994) based on *Paraproutistapseudoalbicosta* Muir, 1915 from Taiwan, as the type species, and falls within the subtribe Lyddina of Zoraidini in the subfamily Otiocerinae (Hemiptera, Fulgoroidea, Derbidae) (Emeljanov 1996; Bourgoin 2025). Then Wu et al. (2003) described four new species from Yunnan Province of China, *N.acutata* Wu & Liang, 2003, *N.bisaccata* Wu & Liang, 2003, *N.furva* Wu & Liang, 2003 and *N.spinellosa* Wu & Liang, 2003, and proposed a new combination, *N.pullata* (Distant, 1911) from India (Assam) and China (Yunnan) (Wu et al. 2003; Bourgoin 2025). It is a relatively small group of Zoraidini, including six species, which are distributed in the Oriental region. All species in the genus have been recorded from China, with only one species found in Taiwan Province, and the rest in Yunnan Province (Yang and Wu 1994; Wu et al. 2003; Bourgoin 2025).

Here, we describe and illustrate two new species, *N.lobata* sp. nov. and *N.quinaria* sp. nov. from southern China, collected on bamboo. As a result, *Neoproutista* now contains eight species, all species recorded from China. A checklist and key based on morphological characteristics are provided to distinguish species, as well as a map of their geographic distributions.

## ﻿Material and methods

Specimens included in the present study were collected using sweeping. All specimens were collected from Guizhou and Fujian Provinces of China. The type specimens examined are deposited in the Institute of Entomology, Guizhou University, Guiyang, Guizhou Province, China (IEGU).

Dry specimens were used for the descriptions and illustrations. Body length was measured from the apex of the vertex to the tip of the forewing. All measurements are in millimeters (mm). The genital segments were removed from the examined specimens and macerated in 10% NaOH, washed in water and transferred to glycerin. Color pictures for adult habitus were obtained using the KEYENCE VHX-6000 system. External morphology and drawings were done under a Leica MZ 12.5 stereomicroscope. Illustrations were scanned with a CanoScan LiDE 200 and imported into Adobe Photoshop v. 6.0 for labeling and plate composition. The distribution map was generated with ArcGIS v. 10.7.

The external morphology terminologies follow Bourgoin (1987), Bourgoin and Huang (1990), Yang and Wu (1994), and Sui and Chen (2019). The standard terminology of venation follows Bourgoin et al. (2015).

## ﻿Taxonomy

**Class Insecta Linnaeus, 1758**

**Order Hemiptera Linnaeus, 1758**

**Infraorder Fulgoromorpha Evans, 1946**

**Superfamily Fulgoroidea Latreille, 1807**

**Family Derbidae Spinola, 1839**

**Subfamily Otiocerinae Muir, 1917**

**Tribe Zoraidini Muir, 1913**

**Subtribe Lyddina Emeljanov, 1995**

### ﻿ Neoproutista

Taxon classificationAnimaliaHemipteraDerbidae

Yang & Wu, 1994

31F2E426-483C-556E-87F2-FDBEF8760C4D

Neoproutista Yang & Wu, 1994: 14; Wu et al. 2003: 463.

#### Type species.

*Paraproutistapseudoalbicosta* Muir, 1915, original designation.

#### Diagnosis.

The distinctive characters proposed by Wu et al. (2003) are modified as follows: head including eyes narrower than pronotum. Vertex triangular or trapezoidal, frons in profile rounded, lateral carinae approximate at middle or apical part, diverging to both ends, area between eyes narrower than width of an eye. Postclypeus shorter than frons, 3-carinate. Antennae with pedicel mostly short, arista terminal. Ocelli present, rudimentary. Mesonotum convex, 3-carinate. Forewing long, longer than widest part (about 3.5:1), with nearly straight inner margin, MP six times branched, MP_1_ four times branched with six terminals, ScP+R, MP, and veins near them often reddish. Hindwing with vein ScP+RA short, MP two or three terminals, CuA two or three terminals. Spinal formula of hind legs 5(6)-5(4~8)-6(4~10). Male terminalia with pygofer narrow; anal tube with basal part form angular processes; gonostyli with basal 1/3 slender, then rapidly expand, inner side with one or two hooked processes; aedeagus asymmetrical, periandrium curved, endosoma more complex. Female terminalia reduced.

#### Host plants.

Bamboo (Poales: Poaceae: Bambusoideae).

#### Distribution.

**Figure 1.**
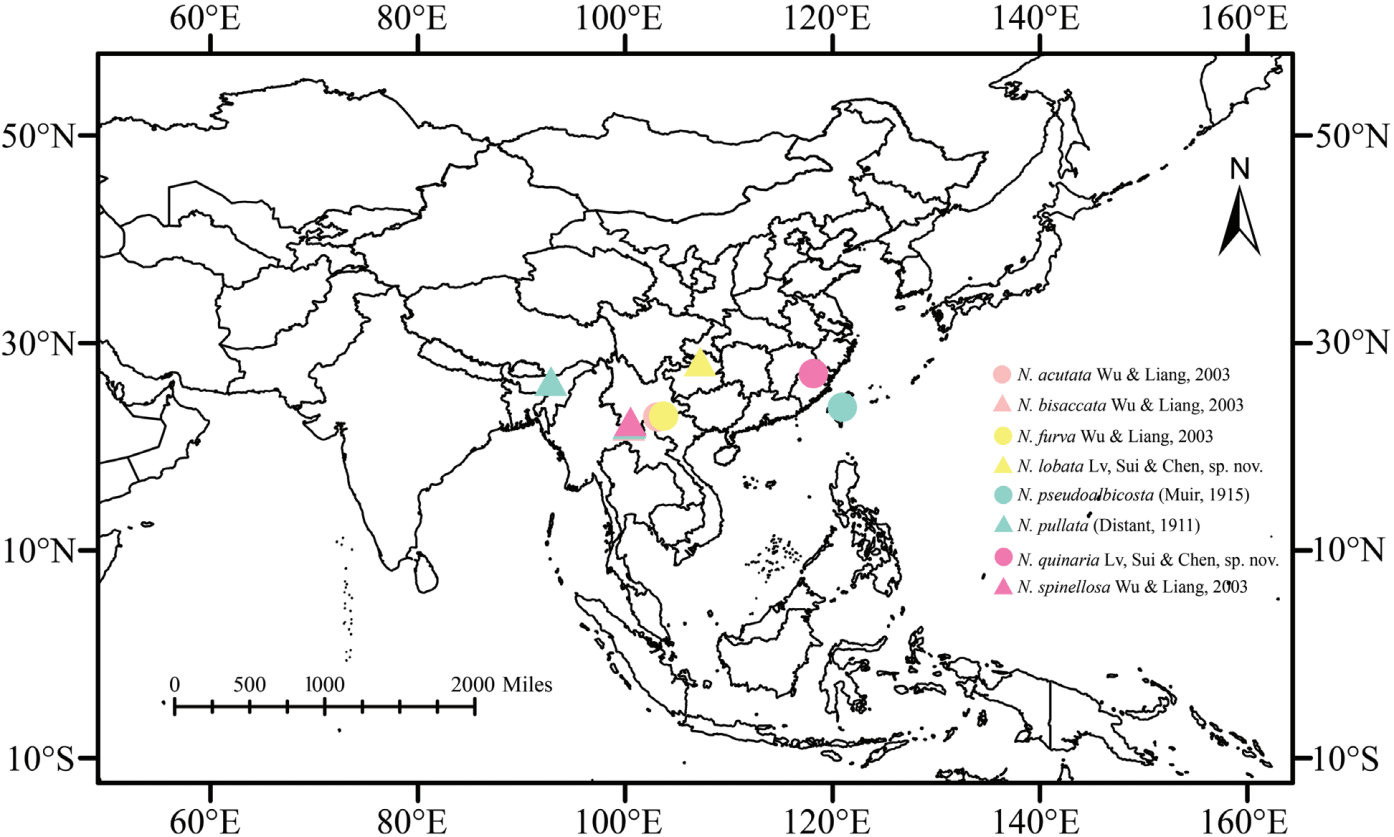
Geographic distributions of species of *Neoproutista* Yang & Wu, 1994.

China (Fujian, Guizhou, Taiwan, Yunnan), India (Assam) (Fig. 1).

##### ﻿Checklist and distributions of species of *Neoproutista* Yang & Wu, 1994

*N.acutata* Wu & Liang, 2003; China (Yunnan)

*N.bisaccata* Wu & Liang, 2003; China (Yunnan)

*N.furva* Wu & Liang, 2003; China (Yunnan)

*N.lobata* Lv, Sui & Chen, sp. nov.; China (Guizhou)

*N.pseudoalbicosta* (Muir, 1915); China (Taiwan)

*N.pullata* (Distant, 1911); China (Yunnan), India (Assam)

*N.quinaria* Lv, Sui & Chen, sp. nov.; China (Fujian)

*N.spinellosa* Wu & Liang, 2003; China (Yunnan)

### ﻿Key to species of *Neoproutista* Yang & Wu, 1994

Modified from Wu et al. 2003.

**Table:** 

1	Gonostyli bifurcate (Wu et al. 2003: figs 49, 52)	***N.pullata* (Distant, 1911)**
–	Gonostyli not bifurcated	**2**
2	Pygofer with dorsocaudal margin protruding caudad (Wu et al. 2003: fig. 37)	***N.acutata* Wu & Liang, 2003**
–	Pygofer with dorsocaudal margin not protruding caudad	**3**
3	Endosoma nearly reaching base of periandrium	**4**
–	Endosoma not reaching base of periandrium	**5**
4	Body blackish brown; anal tube long (Wu et al. 2003: figs 5, 6)	***N.furva* Wu & Liang, 2003**
–	Body reddish brown; anal tube short (Yang and Wu 1994: fig. 3E)	***N.pseudoalbicosta* (Muir, 1915)**
5	Antennae (Figs 6–8) with pedicel long; gonostyli (Figs 11–13) with two hooked processes at inner side	***N.lobata* Lv, Sui & Chen, sp. nov.**
–	Antennae with pedicel short; gonostyli with one hooked process at inner side	**6**
6	Anal tube short; endosoma without spine; gonostyli in lateral view truncate at apex (Wu et al. 2003: figs 16, 17, 19–21)	***N.bisaccata* Wu & Liang, 2003**
–	Anal tube long; endosoma with spine; gonostyli in lateral view angular at apex	**7**
7	Endosoma with small cone-shaped processes at dorsal part; gonostyli asymmetric (Wu et al. 2003: figs 25, 28, 30–32)	***N.spinellosa* Wu & Liang, 2003**
–	Endosoma (Figs 26–28) without small cone-shaped processes at dorsal part; gonostyli symmetric (Figs 23–25)	***N.quinaria* Lv, Sui & Chen, sp. nov.**

### ﻿ Neoproutista lobata

Taxon classificationAnimaliaHemipteraDerbidae

Lv, Sui & Chen sp. nov.

E26A7525-BDFF-57AB-B3A3-BDCC378A1A01

https://zoobank.org/6F05FB8A-0C41-4CD3-8387-7A7098EAA2C3

Figs 2 , 3 , 6–17 , 30 , 31

#### Type materials.

***Holotype***: China • ♂: Guizhou Province, Kuankuoshui National Nature Reserve; 28°14'N, 107°12'E; sweeping, 28 July 2014; Hai-Yan Sun leg.; IEGU. ***Paratypes***: China • 1♂3♀♀; Guizhou Province, Kuankuoshui National Nature Reserve; 28°14'N, 107°12'E; sweeping, 28 July 2014; Hai-Yan Sun, Mei-Na Guo leg.; IEGU. 1♂3♀♀; Guizhou Province, Kuankuoshui National Nature Reserve; 28°14'N, 107°12'E; sweeping, 13 July 2017; Yong-Jin Sui leg.; IEGU.

#### Diagnosis.

**Figures 2–5.**
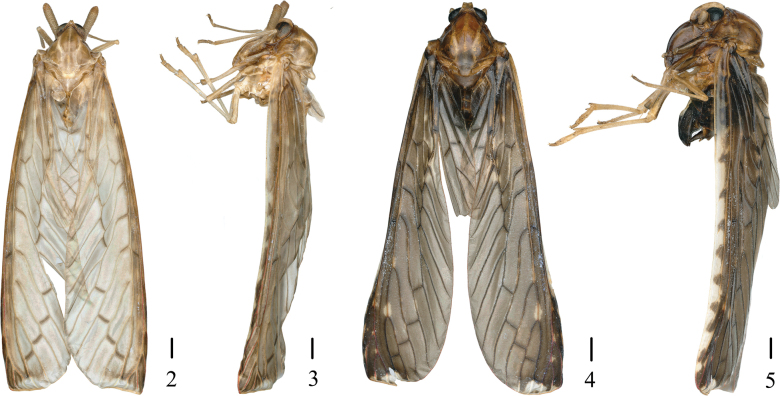
Male habitus (dorsal and lateral views). **2, 3.***Neoproutistalobata* Lv, Sui & Chen, sp. nov.; **4, 5.***Neoproutistaquinaria* Lv, Sui & Chen, sp. nov. Scale bars: 0.5 mm (**2–5**).

**Figures 6–17.**
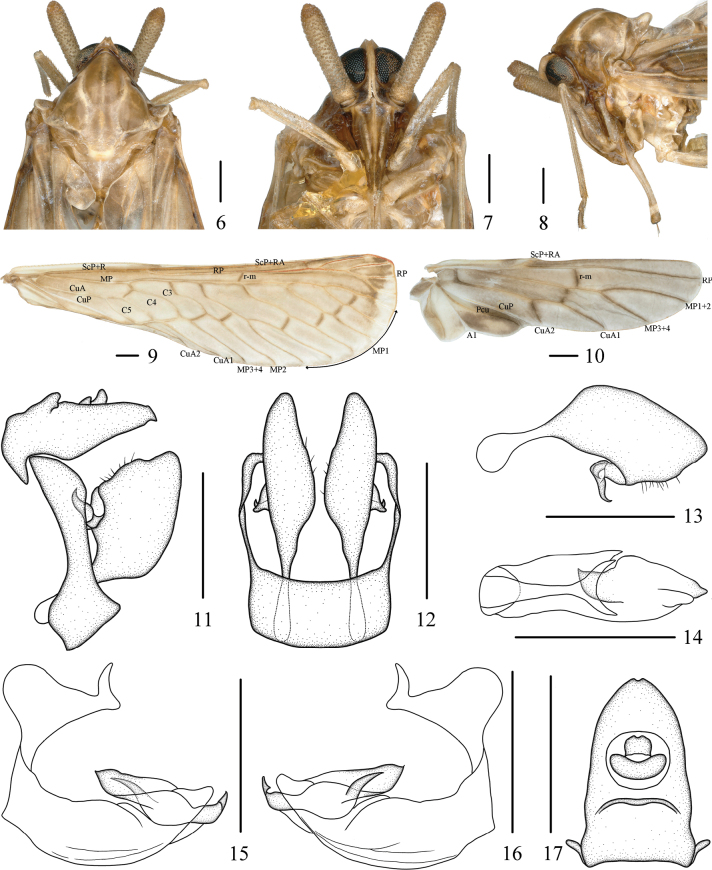
*Neoproutistalobata* Lv, Sui & Chen, sp. nov., male. **6.** Head and thorax, dorsal view; **7.** Frons, ventral view; **8.** Head and thorax, lateral view; **9.** Forewing; **10.** Hindwing; **11.** Terminalia, lateral view; **12.** Terminalia, ventral view; **13.** Gonostyli, inner lateral view; **14.** Aedeagus, dorsal view; **15.** Aedeagus, left lateral view; **16.** Aedeagus, right lateral view; **17.** Anal tube, dorsal view. Scale bars: 0.5 mm (**6–17**).

The salient features of the new species include: general color (Figs 2, 3) light yellowish brown; antennae (Figs 6–8) with pedicel long, about 4.57 times as long as wide; gonostyli (Figs 11–13) symmetrical, inner side with two hooked processes; endosoma (Figs 14–16) about 1/2 length of periandrium, with an apical curly lamellar process at apex, middle part of ventral with a long spinous process. This species is similar to *N.bisaccata* Wu & Liang, 2003, but differs from the latter in: (1) antennae with pedicel long (antennae with pedicel short in *N.bisaccata*); (2) gonostyli with two hooked processes at inner side (gonostyli with one hooked process at inner side in *N.bisaccata*); and (3) endosoma with an apical curly lamellar process at apex (endosoma without an apical curly lamellar process at apex in *N.bisaccata*).

#### Description.

***Measurements*.** Total length: male 9.7–11.5 mm (*N* = 3), female 12.3–12.8 mm (*N* = 6).

***Coloration*.** General color (Figs 2, 3) light yellowish brown. Eyes (Figs 6–8) greyish brown to black. Frons and clypeus (Fig. 7) yellowish brown to tawny. Pronotum (Figs 2, 6) yellowish brown to brown. Mesonotum (Figs 2, 6) with carinae yellowish white. Outer margins of tegula (Figs 2, 6) yellowish white. Forewings (Fig. 9) yellowish white at basal and apical parts, rest yellowish brown to brown, ScP+R red, as shown in Fig. 9. Hindwings (Fig. 10) yellowish brown to brown.

***Head and thorax*.** Head (Figs 2, 3, 6–8) small, including eyes distinctly narrower than pronotum (1:1.61). Vertex (Figs 2, 6) trapezoidal, at base 2.58 times wider than length in midline, apex narrower than base (1:4.44), slightly projecting in front of eyes, anterior margin concave, posterior margin cambered, recessed, lateral carinae slightly developed, median carina absent, disk slightly depressed. Frons (Fig. 7) with lateral carinae developed, more divergent near postclypeus, shorter than clypeus (1:1.6). Clypeus (Fig. 7) with basal part angular on both sides, median carina distinct. Postclypeus (Fig. 7) with three longitudinal carinae. Rostrum (Figs 7, 8) long, extends beyond the coxa of hind leg, apical segment nearly equal in length and width. Eyes (Figs 6–8) with basal part slightly concave in ventral view, ocelli below eyes. Antennae (Figs 6–8) with pedicel long, rod-shaped, about 4.57 times as long as wide. Pronotum (Figs 2, 6) with anterior margin angular on both sides, posterior margin concave in inverted V-shaped, shorter than vertex in midline (1:1.62). Mesonotum (Figs 2, 6, 8) nearly rhomboid, with median and lateral carinae, longer than 6.5 times pronotum and vertex combined, dorsally elevated, in lateral view raised above vertex distinctly. Forewings (Fig. 9) 3.68 times as long as widest point, MP six times branched, MP_1_ four times branched with six terminals. Hindwings (Fig. 10) with vein ScP+RA short, MP two terminals, CuA two terminals. Spinal formula of hind legs 5(6)-7(8)-8(6~10).

***Male terminalia*.** Pygofer (Figs 11, 12) narrow, in lateral view ventral 1/3 nearly quadrangular, dorsal margin sloping, narrowest in the middle; in ventral view oblong. Anal tube (Figs 11, 17) slightly long, in lateral view tapering to ends, basal and apical parts angular, median part of dorsal margin with an angular process; in dorsal view, basal part with an angular process on both sides, lateral margins slightly curved, narrows towards the end, apical margin concave, 1.63 times as long as wide; anal style small, sets at basal 1/3. Gonostyli (Figs 11–13) symmetrical, basal 1/3 slender, then rapidly expand; in lateral view inner and dorsal margins curved, inner side with two hooked processes; in ventral view widest at basal 3/5, outer margin with two hooked processes at basal 3/5. Aedeagus (Figs 14–16) asymmetrical. Periandrium curved, middle part wide relatively. Endosoma more complex, tapering to apex, about 1/2 length of periandrium, with an apical curly lamellar process at apex, middle part of ventral with a long spinous process, slightly curved, directed cephalically.

***Female terminalia.*** Terminalia reduced (Figs 30, 31).

#### Host plant.

Bambusoideae sp. (Poales: Poaceae: Bambusoideae).

#### Distribution.

China (Guizhou Province) (Fig. 1).

#### Etymology.

The species name is derived from the Latin word “*lobata*”, referring to endosoma with an apical curly lamellar process at the apex.

### ﻿ Neoproutista quinaria

Taxon classificationAnimaliaHemipteraDerbidae

Lv, Sui & Chen sp. nov.

93F42A23-7CDC-525D-9666-70AEFD56AFBD

https://zoobank.org/2BFF621F-6889-4953-904C-CD43F244BEF8

Figs 4 , 5 , 18–29 , 32 , 33

#### Type material.

***Holotype***: China • ♂; Fujian Province, Wanmu Forest Provincial Nature Reserve; 27°05'N, 118°15'E; sweeping, 20 May 2012; Jian-Kun Long leg.; IEGU. ***Paratypes***: China • 2♂♂, 4♀♀; Fujian Province, Wanmu Forest Provincial Nature Reserve; 27°05'N, 118°15'E; sweeping, 20 May 2012; Jian-Kun Long and Zhi-Min Chang leg.; IEGU. 11♂♂, 8♀♀; Fujian Province, Wanmu Forest Provincial Nature Reserve; 27°05'N, 118°15'E; sweeping, 27 August 2019; Yong-Jin Sui, Zhi-Cheng Zhou and Xiao-Ya Wang leg.; IEGU.

#### Diagnosis.

The salient features of the new species include: general color (Figs 4, 5) brown; frons (Fig. 19) with lateral carinae divergent from middle to near postclypeus; pygofer (Fig. 23) in lateral view ventral 1/3 nearly triangular; endosoma (Figs 26–28) about 2/3 length of periandrium, with a cone-like process at apex, ventral part with a long process and apical part with five serrations. This species is similar to *N.spinellosa* Wu & Liang, 2003, but differs from the latter in: (1) gonostyli symmetric (gonostyli asymmetric in *N.spinellosa*); (2) endosoma without many small cone-shaped processes at dorsal part (endosoma with many small cone-shaped processes at dorsal part in *N.spinellosa*); and (3) ventral part of endosoma with a long process and apical part with five serrations (ventral part of endosoma without a long process and apical part without five serrations in *N.spinellosa*).

#### Description.

***Measurements*.** Total length: male 10.5–12.8 mm (*N* = 14), female 11.5–13.5 mm (*N* = 12).

**Figures 18–29.**
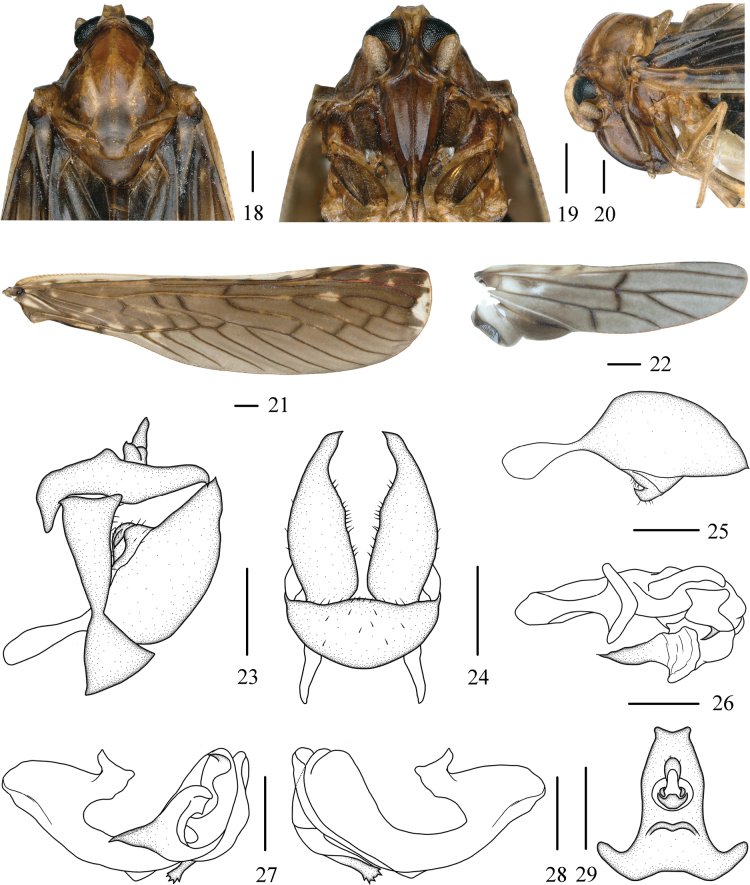
*Neoproutistaquinaria* Lv, Sui & Chen, sp. nov., male. **18.** Head and thorax, dorsal view; **19.** Frons, ventral view; **20.** Head and thorax, lateral view; **21.** Forewing; **22.** Hindwing; **23.** Terminalia, lateral view; **24.** Terminalia, ventral view; **25.** Gonostyli, inner lateral view; **26.** Aedeagus, dorsal view; **27.** Aedeagus, left lateral view; **28.** Aedeagus, right lateral view; **29.** Anal tube, dorsal view. Scale bars: 0.5 mm (**18–29**).

***Coloration*.** General color (Figs 4, 5) brown. Vertex (Figs 4, 18) greyish brown. Eyes (Figs 18–20) black. Frons (Fig. 19) brownish dark medially, lateral carinae brownish dark at basal 1/2. Clypeus (Fig. 19) tawny, a little dark brown at lateral parts. Pronotum (Figs 4, 18) yellowish brown to tawny. Mesonotum (Figs 4, 18) reddish brown to brown, with carinae yellowish white. Forewings (Fig. 21) brown, with yellowish white markings and stripes, costal area yellowish white, ScP+R and MP red to reddish brown at apical to middle part, remaining veins brown mostly, a few light yellowish brown, as shown in Figure 21. Hindwings (Fig. 22) brown, veins blackish brown.

***Head and thorax*.** Head (Figs 4, 5, 18–20) small, including eyes distinctly narrower than pronotum (1:1.58). Vertex (Figs 4, 18) trapezoidal, at base 3.43 times wider than length in midline, apex narrower than base (1:2.01), slightly projecting in front of eyes, anterior margin concave, posterior margin cambered, recessed, lateral carinae slightly developed, median carina absent, disk slightly depressed. Frons (Fig. 19) with lateral carinae developed, divergent from middle to near postclypeus, shorter than clypeus (1:2.17). Clypeus (Fig. 19) with basal part angular on both sides, median carina distinct. Postclypeus (Fig. 19) with three longitudinal carinae. Rostrum (Fig. 19) long, extends beyond the coxa of hind leg, apical segment slightly longer than width. Eyes (Figs 18–20) with basal part slightly concave in ventral view, ocelli below eyes. Antennae (Figs 18–20) with pedicel short, elliptic, about 1.9 times as long as wide. Pronotum (Figs 4, 18) with anterior margin angular on both sides, posterior margin concave in inverted V-shaped, shorter than vertex in midline (1:1.63). Mesonotum (Figs 4, 18, 20) nearly rhomboid, with median and lateral carinae, longer than 5.63 times pronotum and vertex combined, dorsally elevated, in lateral view raised above vertex distinctly. Forewings (Fig. 21) 3.98 times as long as widest point, MP six times branched, MP_1_ four times branched with six terminals. Hindwings (Fig. 22) with vein ScP+RA short, MP two terminals, CuA two terminals. Spinal formula of hind legs 5-6-3(4).

**Figures 30–33.**
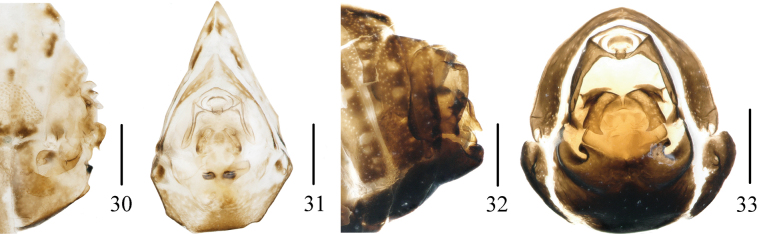
Female terminalia (lateral and caudal views). **30, 31.***Neoproutistalobata* Lv, Sui & Chen, sp. nov.; **32, 33.***Neoproutistaquinaria* Lv, Sui & Chen, sp. nov. Scale bars: 0.5 mm (**30–33**).

***Male terminalia*.** Pygofer (Figs 23, 24) narrow, basal 1/3 narrowest, in lateral view ventral 1/3 nearly triangular, dorsocaudal angle slightly produced; in ventral view widest in the middle. Anal tube (Figs 23, 29) slightly long, in lateral view tapering to ends, basal part obtuse, apical part cuspidal, median part of dorsal margin angularly produced; in dorsal view, basal part with a long stout process on both sides, lateral margins slightly curved, narrows towards the end, apical margin concave, form a small process on both sides; anal style small, sets at basal 1/2. Gonostyli (Figs 23–25) symmetrical, basal 1/3 slender, then rapidly expand; in lateral view tapering to apex, apical part cuspidal, inner and dorsal margins curved, inner side with a hooked process; in ventral view widest at middle part, tapering to apex. Aedeagus (Figs 26–28) asymmetrical. Periandrium curved, middle part narrow. Endosoma more complex, tapering to apex, about 2/3 length of periandrium, with a cone-like process at apex, directed cephalically, ventral part with a long process and apical part with five serrations, directed ventrocephalad.

***Female terminalia*.** Terminalia reduced (Figs 32, 33).

#### Host plant.

*Phyllostachysheteroclada* Oliv. (Poales: Poaceae: Bambusoideae).

#### Distribution.

China (Fujian Province) (Fig. 1).

#### Etymology.

The species name is derived from the Latin word “*quinaria*”, referring to the ventral part of the endosoma with a long process and five serrations apically.

## ﻿Discussion

In this paper, we describe two new species from China and place them in the genus *Neoproutista*. The discovery of these species expands our understanding of the morphology and biogeography of the genus. According to Yang and Wu (1994) and Wu et al. (2003), the main distinguishing features of this genus are as follows: postclypeus shorter than frons, 3-carinate; antennae with pedicel extremely short, arista terminal; and forewing with nearly straight inner margin, longer than widest part (about 3.5:1), MP vein six times branched, third one branched. However, our study of *N.lobata* sp. nov. reveals that its antennae do not conform to these characteristics, as the pedicel is long and extends beyond the eyes (Figs 2, 3, 6–8). Therefore, we propose that the generic diagnosis for antennae in this genus should be modified to indicate that the antennae are mostly short.

The genus *Neoproutista* was previously known to contain only six species. However, based on the findings of this research, the total number of species within the genus has been updated to eight. At present, all species are distributed in the Oriental region (Fig. 1), and the genus is especially speciose in China, where all species recorded to date appear to be endemic to China. There is little doubt that the highly diverse natural conditions in China will lead to the discovery of additional new species within this genus in the future.

The host plants of the Derbidae family have been documented across a range of orders, including Alismatales, Arecales, Asparagales, Asterales, Boraginales, Cyatheales, Ericales, Fabales, Fagales, Gentianales, Laurales, Liliales, Malpighiales, Malvales, Pandanales, Pinales, Piperales, Podocarpales, Poales, Rosales, Sapindales, Scrophulariales, Solanales, Theales, and Zingiberales (Muir 1913; Fennah 1952; Wilson 1987; Löcker et al. 2009; Zelazny and Webb 2011; Dollet et al. 2020; Campodonico 2021; Sui et al. 2023; Chen and Yang 2024; Chen et al. 2024; Bourgoin 2025), but no information has been reported on the host plants of *Neoproutista*. Based on our observations during field trips, these two new species, *N.lobata* Lv, Sui & Chen, sp. nov. and *N.quinaria* Lv, Sui & Chen, sp. nov., from southern China, were collected on bamboo (Poales, Poaceae, Bambusoideae), which might be the plant on which they feed.

## Supplementary Material

XML Treatment for Neoproutista

XML Treatment for Neoproutista lobata

XML Treatment for Neoproutista quinaria

## ﻿Additional information

### Data availability

All of the data that support the findings of this study are available in the main text.
